# Population Pharmacokinetics of Rituximab in Pediatric Patients With Frequent-Relapsing or Steroid-Dependent Nephrotic Syndrome

**DOI:** 10.3389/fphar.2021.725665

**Published:** 2021-09-02

**Authors:** Yewei Chen, Qian Shen, Min Dong, Ye Xiong, Hong Xu, Zhiping Li

**Affiliations:** ^1^ Department of Pharmacy, Children’s Hospital of Fudan University, Shanghai, China; ^2^ Department of Nephrology, Children’s Hospital of Fudan University, Shanghai, China; ^3^ Division of Clinical Pharmacology, Cincinnati Children’s Hospital Medical Center, Cincinnati, OH, United States; ^4^ Department of Pediatrics, University of Cincinnati, Cincinnati, OH, United States

**Keywords:** rituximab, pharmacokinetics, children, nephrotic syndrome, dosing

## Abstract

**Objectives:** Rituximab is frequently used off-label for the treatment of frequent-relapsing nephrotic syndrome (FRNS) or steroid-dependent nephrotic syndrome (SDNS), but the relapse rate remained high and the dosing regimen varied widely. The objective of this study was to characterize rituximab pharmacokinetics (PK) in pediatric patients with FRNS/SDNS, and to investigate the differences in rituximab PK between patients with FRNS/SDNS and other disease populations.

**Methods:** Fourteen pediatric patients received rituximab for FRNS/SDNS treatment were enrolled in a prospective, open-label, single-center PK study. A population PK model of rituximab was developed and validated, and PK parameters were derived for quantitative evaluation.

**Results:** A two-compartment PK model best described the data. Body surface area was the most significant covariate for both central clearance (CL) and apparent central volume of distribution (V_1_). Patients with FRNS/SDNS exhibited a clinically relevant increase in rituximab CL compared to patient population with non-Hodgkin’s lymphoma (NHL).

**Conclusion:** This pilot study indicated that higher doses or more frequent regimens of rituximab may be required for optimal therapeutic effects in patients with FRNS/SDNS. Further clinical studies with more patients are warranted to confirm this result.

## Introduction

Primary nephrotic syndrome (NS) is the most common chronic glomerular disease in children (C (1978). Nephrotic s, 1978). Despite the fact that approximately 80% of these children are recognized as steroid-sensitive nephrotic syndrome (SSNS) (C (1981). The primary, 1981), up to 50% of these SSNS patients will develop to frequent-relapsing nephrotic syndrome (FRNS) or steroid-dependent nephrotic syndrome (SDNS) (Schulman et al., 1988). These children often experience steroid toxicities and complications of immunosuppression (Park and Shin, 2011), suggesting that there is an urgent need to develop new treatments for children with FRNS/SDNS. Rituximab, a chimeric anti-CD20 monoclonal antibody, originally approved for the treatment of non-Hodgkin’s lymphoma (NHL), later granted for the treatment of chronic lymphocytic leukemia (CLL) and several autoimmune diseases including rheumatoid Arthritis (RA), granulomatosis with polyangiitis and Microscopic Polyangiitis. Recently, rituximab has been increasingly used off-label for the management of FRNS and SDNS. Case reports and randomized controlled trial (RCT) studies in children with FRNS/SDNS have demonstrated promising results (Ravani et al., 2011; Ravani et al., 2015; Basu et al., 2018). Rituximab appears to be effective for FRNS/SDNS to maintain remission, reduce or discontinue steroids and immunosuppressants. Findings were confirmed by a multicenter double-blind RCT conducted in Japan, which contributed to Japanese government approval for the use of rituximab in patients with FRNS/SDNS (Iijima et al., 2014).

Although more and more studies demonstrated efficacy of rituximab in maintaining remission in FRNS/SDNS, the relapse rate remained high and the optimal dosing schedule has not been established. In a multicenter prospective study reported by Kamei et al. only three patients (25%) with refractory SDNS did not have a relapse and the disease was kept in remission for more than 1 year (single dose at 375 mg/m^2^) (Kamei et al., 2009). Kemper et al. reported that 37 (70%) patients with SDNS remained in remission after 1 year and 12 out of 29 (41%) patients with SDNS remained in remission for >2 years who were treated with rituximab (1–4 doses at 375 mg/m^2^) (Kemper et al., 2012). In an RCT study conducted by Ravani et al., 46 patients with steroid and calcineurin inhibitor-dependent nephrotic syndrome treated with rituximab (1–5 doses at 375 mg/m^2^) were included, 48% after the first infusion and 37% after subsequent infusions remained in remission within 6 months, whereas 1- and 2-years-remission probabilities were, respectively, 20 and 10% (Ravani et al., 2013).

So far, very few studies have studied the PKs of rituximab in patients with NS. Unlike small molecules, rituximab is not subject to classical drug metabolism or renal elimination. After i.v. administration, rituximab rapidly binds to the CD20 antigen expressed on the surface of B cells in the peripheral blood, bone marrow and lymph nodes (Keating, 2010). Rituximab clearance is the sum of linear non-specific clearance and non-linear specific clearance, including non-specific Fcγ receptors-independent endocytosis, non-specific proteolysis which takes place in the liver and other organs, and specific target-mediated drug disposition (TMDD) (Golay et al., 2013). Rituximab are too large to be filtered by the kidneys and are not eliminated in the urine, except in pathologic condition. One study has shown that a significant amount of rituximab is lost in the urine in a pediatric patient with NS during a period of heavy proteinuria (Counsilman et al., 2015). Thus, the PK of rituximab in children with FRNS/SDNS may be different from that in other patient populations such as NHL, CLL, and RA. The aim of this study was to perform a population PK study of rituximab to characterize rituximab PK in pediatric patients with FRNS/SDNS and to investigate the differences in rituximab PK between patients with FRNS/SDNS and other disease populations.

## Methods

### Patients and Data Collection

A prospective, open-label, single-center PK study was conducted in Nephrology Department at Children’s Hospital of Fudan University between January and July 2017. Patients younger than 16 years with diagnosed FRNS or SDNS (primary nephrotic syndrome), with negative proteinuria, being treated with rituximab, were eligible for enrollment in the study. Patients were excluded if they were tested positive for hepatitis B or C, or tuberculosis, or human immunodeficiency virus, or had other active infections. The study protocol was approved by the Ethics Committee of Children’s Hospital of Fudan University. All study participants were enrolled after obtaining written permission (informed consent) from each child’s parents or legal guardians.

The following demographic and laboratory factors were collected for all patients at each visit: gender, proteinuria (PRO), body weight (BW), height (HT), body surface area (BSA), age, age at onset, NS duration, serum albumin (ALB), serum total cholesterol (TCH), serum creatinine (Scr), creatinine clearance rate (CLCR, where *k* = 0.413 for children 1–13 years old and 0.70 for males between the ages of 13 and 20) (Schwartz et al., 2009), blood urea nitrogen (BUN), cystatin C (CysC), and proteinuria-to-creatinine ratio (PRO/CR).

### Administration of Treatment

Rituximab was administered intravenously at a dose of 375 mg/m^2^, with a maximum of 500 mg once weekly for a maximum of 2 weeks. For patients with recurrence of proteinuria, rituximab was administered only once. All patients were premedicated with methylprednisolone in order to minimize side effects associated with the treatment regimen. After remission, the dose of immunosuppressive agents was gradually reduced for 3–6 months. For the first dose, rituximab was administered intravenously started at 50 mg/h during the first 30 min. If no signs of infusion-related side effects were observed, a following and the second rituximab dose was administered at the same infusion rate. The median infusion duration was 4.75 h (ranged from three to 6.5 h). If hypersensitivity or toxicity occurred, the infusion was temporarily slowed or interrupted and resolved with supportive care. The infusion was continued at 25 mg/h upon improvement of patient symptoms.

### Laboratory Analysis

For each patient, 11 serum samples (1.5 ml) were planned to collect for PK analysis: at predose, the end of infusion, and 24, 48, 72, and 168 h after the first infusion, then at the end of the second infusion, and 14, 30, 60, and 90 days after the last infusion. However, due to the long follow-up and randomness of outpatient visits most of the planned points after the last infusion were not collected.

Blood was drawn into K_2_ EDTA tube and allow at room temperature (RT) for 30 min. Samples were centrifuged at 5000 RPM for 10 min at RT and stored at −20°C until analysis. Rituximab concentrations were measured by enzyme-linked immunosorbent assay method (ELISA), based on rituximab ELISA kit (SHIKARI^®^, MATRIKS BIOTECHNOLOGY CO., LTD.). Standards (from 0 to 300 ng/ml), low/high level control and diluted samples (1/10,000) were added and the plates were incubated for 1 h at room temperature. After incubation, the wells were washed. Horse radish peroxidase conjugated probe was added and the plates were incubated for 1 h at room temperature. Following incubation wells were washed and the chromogen substrate tetramethylbenzidine was added until the appropriate color had developed. The reaction was terminated absorbance at 450 nm was measured with a multi-plate reader (Synergy 2, BioTek, Winooski, VT, United States). Diluted samples were determined using the standard curve. The lower limit of quantification (LLOQ) was 3 ng/ml. The level below the rituximab LLOQ (2 samples) was treated as missing. The intra-assay and inter-assay expressed as CV were both less than 15%.

### Pharmacokinetic Analysis

#### Model Development

A population PK analysis of rituximab was performed with the NONMEM program (version VII, Icon Development Solutions, Ellicott City, MD, United States) in conjunction with Wings for NONMEM. The first order conditional estimation (FOCE) method with interaction option was used throughout the model-building procedure. Two linear models, a one- and two-compartmental PK model were fit to rituximab concentration-time data. On the basis of the best linear model, TMDD was modelled as non-linear clearance, approximated by time-varying function or Michaelis-Menten elimination. Inter-individual variability was described by an exponential model. Inter-individual variances that could not be estimated properly were fixed to 0. Additive, proportional and combined residual error models were evaluated for residual variability, respectively.

The demographic information was used to perform an initial selection of covariates. The association between demographic factors and pharmacokinetic parameters was evaluated by graphical exploration followed by testing within NONMEM with a stepwise covariate modelling procedure. Among covariate effects considered for inclusion were patients’ gender, BW, HT, BSA, age, age at onset, NS duration, ALB, TCH. As disease state may affect the PK of rituximab, covariates reflecting renal function such as Scr, CLCR, BUN, CysC, PRO, and PRO/CR were also tested. For inclusion of continuous covariates, exponential models were investigated. Categorical covariates were included using a category variable equation. The selection of covariates was determined using a forward selection process and a backward elimination process. Nested models were statistically compared using a likelihood ratio test on the differences in the objective function value (OFV). A reduction in OFV of 3.84 (*p* < 0.05) for forward inclusion and an increase in OFV of 6.63 (*p* < 0.01) for backward elimination were the criteria for retaining a covariate in the model. Non-nested models were compared by Akaike information criteria (AIC) calculated by Pirana software (ver. 2.7.1; Pirana Software and Consulting BV, http://www.pirana-software.com/).

#### Model Evaluation

Models were evaluated graphically using goodness-of-fit diagnostic plots: observed concentrations (DV) versus population predicted concentrations (PRED), DV versus individual predicted concentrations (IPRED), conditional weighted residuals (CWRES) versus time (TIME), CWRES versus PRED.

The stability of the final model was assessed by non-parametric bootstrap (Ette et al., 2003). One thousand data sets were generated by randomly resampling from the original data set. The values of bootstrap estimates with 95% confidence intervals were compared with those estimated from the original dataset. It could be proved that the model was stable if the values of parameters were not significantly different.

A visual predictive check (VPC) was performed to evaluate the predictive performance of the model (Holford, 2005). A total of 1,000 replicates were simulated using the final model estimates. The median, 5th and 95th percentiles of the simulated concentrations were constructed and compared with the observed concentrations. The model was deemed precise if the observed concentrations were appropriately distributed within the 5th–95th prediction interval.

## Results

### Patient Characteristics

**FIGURE 1:**
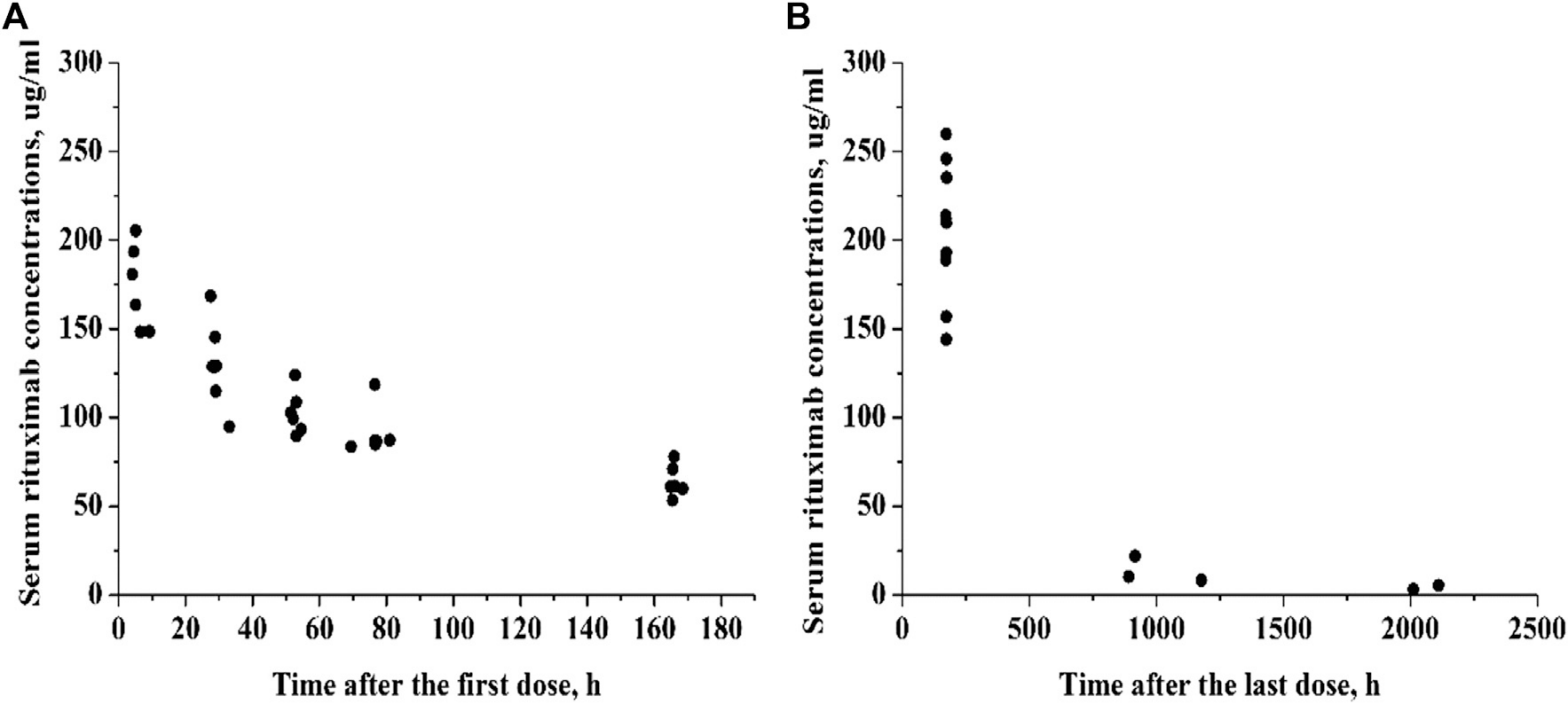
Individual serum rituximab concentration-time profiles. **(A)** Concentration plots after the first dose. **(B)** Concentration plots after the last dose.

The study population consisted of 14 pediatric patients with FRNS/SDNS. Five patients presented FRNS, 7 had SDNS, and 2 FRNS/SDNS. There were 8 patients with minimal change disease (MCD), three patients with focal segmental glomerulosclerosis (FSGS), and three without renal biopsy. Eleven out of the 14 patients received two infusions of rituximab, three children received only once because of the recurrence of proteinuria. Their clinical characteristics are shown in Table 1. In total, 72 serum rituximab concentrations were available for pharmacokinetic analysis. Individual serum rituximab concentration plots (concentration versus time) are shown in Figure 1.

**TABLE 1:** Characteristics of patients.

Characteristic	Mean (±SD)	Median	Range
No. of patients/samplings	14/72		
Gender (Boys/Girls)	13/1		
PRO (Positive/Negative)	3/11		
BW (kg)	32.5 (22.0)	23.2	15.0–96.5
HT (cm)	125.3 (25.4)	113.5	92.0–165.0
BSA (m^2^)	1.0 (0.4)	0.9	0.6–2.1
Age(y)	8.3 (4.7)	6.8	3.0–15.6
Age at onset (y)	5.0 (3.4)	3.9	1.4–13.3
NS duration (m)	40.1 (28.1)	31.5	9.0–104.0
ALB (g/L)	35.6 (6.3)	36.4	23.3–42.9
TCH (mmol/L)	6.2 (1.9)	5.9	3.4–11.2
SCR (µmol/L)	33.4 (13.2)	30	20–62
CLCR (mL/min/1.73 m^2^)	169.5 (29.3)	165.4	118.9–217.2
BUN (mmol/L)	4.7 (1.5)	4.8	2.8–8.2
CysC (mg/L)	0.9 (0.2)	0.9	0.6–1.2
PRO/CR	0.3 (0.5)	0.1	0.04–1.68

*PRO, proteinuria; BW, body weight; HT, height; BSA, body surface area; NS, nephrotic syndrome; ALB, serum albumin; TCH, serum total cholesterol; Scr, serum creatinine; CLcr, Creatinine clearance rate,; BUN, blood urea nitrogen; CysC, cystatin C; PRO/CR, proteinuria-to-creatinine ratio.

### Population PK Modeling

Preliminary analysis for the linear model showed that OFV of one- and two-compartment models were 554.96 and 533.11, respectively. A two-compartment model resulted in a better fit to describe rituximab concentrations based on the change of OFV (*δ* = 21.85) and diagnostic plots. The two-compartment models with time-dependent elimination (AIC = 613.847), and with Michaelis-Menten elimination (AIC = 615.118) did not improve model performance compared to the linear two-compartment model (AIC = 575.117). Therefore, the two-compartment model was selected as the structural model. Residual variability was best described by a proportional error model.

Of all covariate relationships tested, we observed a significant association of CL with BSA, and V_1_ with BSA. The inclusion of BSA in CL was associated with a decrease in OFV from 533.11 to 520.45 and a decrease in the inter-individual variability of CL from 56.0 to 32.9%. The inclusion of BSA as a covariate of V_1_ was associated with a decrease in OFV from 520.45 to 511.71 and a decrease in the inter-individual variability of V_1_ from 39.7 to 26.7%. The effect of Scr, CLCR, BUN, CysC, PRO, and PRO/CR were all not found to be significant when included as covariates. Parameters of the final model are summarized in Table 2.

**TABLE 2:** Parameter estimates of rituximab final model and bootstrap validation.

	Final model	Bootstrap *n* = 1,000	
Parameter	Population estimate (RSE (%))	Median	95% CI
CL (ml/h)	CL = θ1*(BSA/0.9)^θ5^		
θ_1_	8.69 (11.9)	8.75	6.42–10.9
θ_5_	1.26 (16.3)	1.26	0.787–1.91
V_1_ (L)	V1 = θ2*(BSA/0.9)		
θ_2_	1.86 (7.7)	1.84	1.56–2.11
Q (ml/h)	Q = θ3		
θ_3_	7.5 (31.5)	7.77	4.89–21.0
V_2_ (L)	V2 = θ4		
θ_4_	1.9 (16.7)	1.86	1.05–2.5
Inter-individual variability (%)			
IIV_CL_	32.9 (48.5)	30.9	15.6–51.4
η_CL_-shrinkage (%)	11.6		
IIV_V1_	26.7 (37.4)	25.9	14.6–34.5
η_V1_-shrinkage (%)	4.01		
Residual error model (%)			
Proportional	15.2 (18.7)	14.6	11.4–17.4
ε-shrinkage (%)	15.9		

*CL, central clearance; V_1_, central volume of distribution; Q, inter-compartment clearance; V_2_, peripheral volume of distribution; IIV, inter-individual variability.

*RSE (%), relative standard error; CI, confidence interval.

### Model Evaluation

**FIGURE 2:**
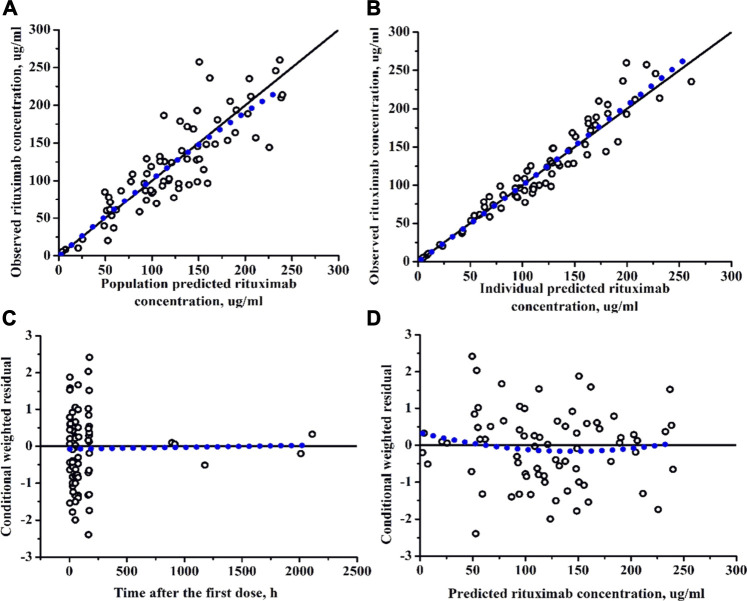
Diagnostic plots of rituximab final PK model. **(A)** The observed versus population-predicted concentration. **(B)** The observed versus individual-predicted concentration. **(C)** Conditional weighted residual versus time. **(D)** Conditional weighted residual versus the predicted concentration.

**FIGURE 3:**
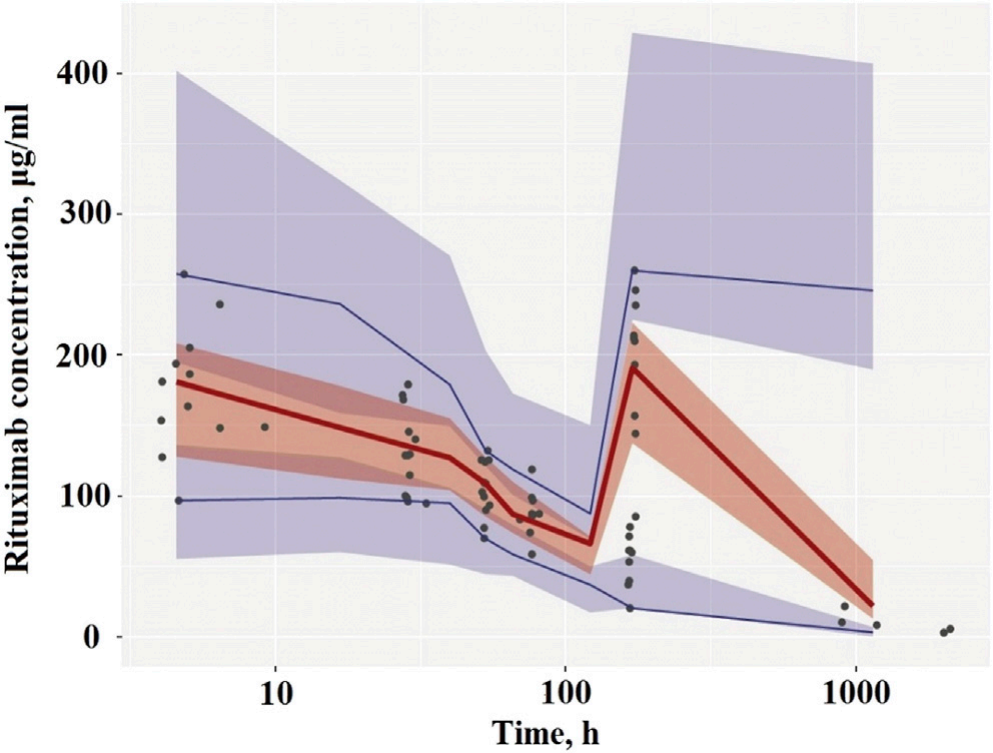
Visual predictive check of the final PK model. **(A)** Plots after the first dose. **(B)** Plots after the last dose. Circles represent the observed concentrations. Solid lines represent the observed median, 5th and 95th percentiles of observed profiles. The shaded areas correspond to the simulation-based 95% confidence intervals.

Diagnostic plots for the final rituximab model showed a good model fit (Figure 2). The results of 1,000 bootstrap replicates for rituximab are summarized in Table 2. The number of runs with successful convergence was 923. The median parameter estimates from the bootstrap procedure were very close to the values of the final population model. In addition, the parameters from the bootstrap procedure followed a normal distribution and contained all of the parameter estimates from the final population model. The results indicate that the estimates for the population PK parameters in the final model were precise and that the model was stable. The VPC plot for the final model is presented in Figure 3. The observed median, 5th and 95th percentiles were in good agreement with the simulated median, 5th and 95th percentiles, indicating the good predictive performance of the final model.

## Discussion

To establish more effective dosing regimens, we investigated rituximab disposition and the impact of covariates on PK in pediatric patients with FRNS/SDNS in an open prospective study using a population approach. To the best of our knowledge, this is the first population PK model for rituximab in pediatric patients with FRNS/SDNS. Thirteen PK models of rituximab have been reported in different disease populations: in patients with NHL (Regazzi et al., 2005; Blasco et al., 2009; Müller et al., 2012; Gota et al., 2016; Tout et al., 2017a; Rozman et al., 2017; Candelaria et al., 2018; Ternant et al., 2019), in patients with chronic lymphocytic leukemia (CLL) (Li et al., 2012; Tout et al., 2017b), in patients with rheumatoid arthritis (Ng et al., 2005; Lioger et al., 2017) and in patients under plasmapherisis (Puisset et al., 2013). Among these studies, rituximab PK was described as a two-compartment model (Ng et al., 2005; Regazzi et al., 2005; Blasco et al., 2009; Li et al., 2012; Müller et al., 2012; Puisset et al., 2013; Gota et al., 2016; Tout et al., 2017a; Tout et al., 2017b; Lioger et al., 2017; Rozman et al., 2017; Candelaria et al., 2018; Ternant et al., 2019). Nonlinear specific clearances corresponding to TMDD were reported in 4 studies, in lymphomas and CLL (Li et al., 2012; Tout et al., 2017b; Rozman et al., 2017; Ternant et al., 2019), but was not described in rheumatoid arthritis despite the influence of CD20 antigen mass on rituximab clearance (Ng et al., 2005; Lioger et al., 2017). In the present study, although the PK profile of rituximab displayed a biphasic decline which is generally characterized by a two-compartment model, we still tested time-varying function and Michaelis-Menten elimination. However, the addition of nonlinear elimination did not improve model performance. The absence of nonlinear PK in patients with FRNS/SDNS may be explained by low target amount compared to lymphomas and CLL, and TMDD did not contribute clearance significantly.

The only covariate included in the final model was body surface area (covariate of CL and V_1_), which decreased OFV and inter-individual variability. The results were similar to the findings of previous publications in other disease populations, in which body surface area, body weight or age were the most significant covariates for CL, and body surface area was the most significant covariate for V_1_ (Ng et al., 2005; Blasco et al., 2009; Li et al., 2012; Müller et al., 2012; Tout et al., 2017a; Tout et al., 2017b; Lioger et al., 2017; Candelaria et al., 2018). Müller et al. found that clearance was significantly reduced in the female patients and was 1.5-fold faster in male patients (Müller et al., 2012). Gender was one of the most significant covariates for both CL and V_1_ in the study by Ng et al. (2005). Similarly, Rozman et al. and Lioger et al. also reported that V_1_ was higher in males (Lioger et al., 2017; Rozman et al., 2017). But in our present study, the covariate effect of gender on rituximab PK was not identified probably due to the limited number of female patients. As disease state may affect the PK of rituximab, covariates reflecting renal function and proteinuria were considered in the current study and none of them had powerful effects on rituximab clearance.

The final PK parameters of rituximab were compared with those reported in previous studies (Table 3). Linear non-specific clearance from the central compartment in patients with FRNS/SDNS was much higher than patients with lymphomas reported by the overwhelming majority of studies (Regazzi et al., 2005; Blasco et al., 2009; Müller et al., 2012; Gota et al., 2016; Candelaria et al., 2018; Ternant et al., 2019), and approximately 3 times higher compared to CLL patients (Li et al., 2012; Tout et al., 2017b), which is in line with the previous publication that patients with membranous nephropathy exhibited an increased rituximab clearance from the central compartment (Fogueri et al., 2019). One possible explanation is that rituximab is filtered by the kidneys and eliminated in the urine due to kidney impairment. Counsilman et al. reported that the serum half-life of rituximab was extremely short and at least 25% of rituximab was lost in the urine in a pediatric patient with steroid-resistant NS during relapse (Counsilman et al., 2015). In our current study, all the patients with FRNS/SDNS were in remission and none of the renal function indicators was found to have a significant effect on PK parameters when included as a covariate. We did not measure urinary rituximab levels directly because the kit we used was only available in serum and plasma. We are currently developing a method to determine the concentrations of rituximab in urine. In future studies, we will examine whether rituximab could be eliminated in the urine. Also, other possible mechanisms need to be explored to explain the observed increased linear clearance.

**TABLE 3:** Exposure parameters of rituximab in population pharmacokinetic studies.

Study	Year	Type of patients	Linear elimination (ml/h/m^2^)	Nonlinear elimination (ml/h/m^2^)	Central volume of distribution (L/m^2^)
This study		FRNS/SDNS	9.7 (32.9% CV)	—	2.0 (26.7% CV)
Regazzi	2005	FL	5.1 (3.8–8.1)	—	1.75 (1.56–2.13)
Blasco	2009	FL or DLBLC	2.4 (73.9% CV)	—	1.77 (12.9% CV)
Ternant	2019	FL or DLBLC	5.3 (42% CV)	Not estimated (TMDD)	1.6 (19% CV)
Müller	2012	DLBLC	5.2 (4.5–6)	—	2.2 (1.9–2.4)
Rozman	2017	DLBLC	10.5 (ml/h) (19.7% CV)	11.6 (ml/h) (Time-varying)	4.14 (L) (16.6% CV)
Tout	2017	DLBLC	12.4 (48.2% CV)	—	3.5 (28.7% CV)
Candelaria	2018	DLBLC	7.3 (24.7% CV)	—	1.9 (14.2% CV)
Gota	2016	DLBLC	5.89 (ml/h) (22.3% CV)	—	0.95 (L) (48.8% CV)
Li	2012	CLL	3.8 (47% CV)	27.8 (Time-varying)	2.2 (40% CV)
Tout	2016	CLL	3.0 (29.9% CV)	140.4 (TMDD)	1.6 (18% CV)
Ng	2005	RA	6.2 (3.4% CV)	—	2.9 (2.1% CV)
Lioger	2017	RA	13.0 (29% CV)	—	2.6 (22% CV)
Puisset	2013	Plasmapheresis	3.6 (35.6% CV)	—	1.4 (20.5% CV)

*FRNS/SDNS, frequent-relapsing nephrotic syndrome/steroid-dependent nephrotic syndrome; FL, follicular lymphoma; DLBCL, diffuse large B-cell lymphoma; CLL, chronic lymphocytic leukemia; RA, rheumatoid arthritis.

*TMDD, target-mediated drug disposition.

There is no unified dosing regimen of rituximab in pediatric patients with FRNS/SDNS. In Japan, 375 mg/m^2^ weekly for 4 weeks has been approved for the treatment of complicated FRNS/SDNS in both adult and pediatric patients (Iijima et al., 2014). In the United Kingdom, a national policy statement recommends that patients weighing <50 kg take two doses of 750 mg/m^2^ on day 1 and 15 (Specialised Commissioning, 2015). When rituximab was first introduced to treat pediatric NS, using 375 mg/m^2^ weekly for 4 weeks, which is borrowed from the protocol for the treatment of NHL. Subsequently, many dosing regimen strategies have been reported, ranging from 100 to 750 mg/m^2^ per dose, with administration of 1–5 doses. Maxted et al. in their study compared 7 different dosing strategies in 60 pediatric patients with FRNS/SDNS. They showed that a single low dose of 375 mg/m^2^ has a similar outcome in maintaining remission compared to higher doses (2 doses at 750 mg/m^2^) at 6 and 12 months, but this is not the case at 2 years (Maxted et al., 2019). In contrast, Hogan et al. reported a study involving 61 SDNS patients receiving three different rituximab regimens (single dose at 100 mg/m^2^, single dose at 375 mg/m^2^, and 2 doses at 375 mg/m^2^, respectively). The authors concluded a lower dose is associated with a higher risk of relapse (Hogan et al., 2019). It should be noted that both of the studies had small number of patients. An enhanced clearance of rituximab reported in our study indicates that a dose of 375 mg/m^2^, once weekly for 2 weeks may not be enough for controlling FRNS/SDNS in pediatric patients. Increasing rituximab doses or more frequent regimens may be required for optimizing therapeutic effects in this population. Rituximab is generally well-tolerated in most published studies of its use in FRNS/SDNS. In the present study, two children have mild infusion-related reactions, which resolve with slowing the infusion rate.

Some limitations of this study should be considered. First, the PK study was limited in size to 14 pediatric patients who were predominantly male. The relatively large numbers of subjects may improve the precision of parameters. Secondly, most of the planned sampling points after the last infusion were lost due to the randomness of outpatient visits and long-term follow-up. Population PK analysis can be based on “sparse” (few observations/subject) and “unbalanced” data (Mould and Upton, 2013), which are frequent features in pediatrics because of ethical and practical issues. This method is the application of a model to describe data that arise from more than one individual. The process does not require that each individual provides “rich” data to characterize completely their own PK profile, which allows borrowing of information between individuals to fill in gaps in the PK profiles (Duffull et al., 2011). In the present study, the relative standard error (RSE%) for the primary PK parameters CL and V_1_ are low (<15%), indicating good certainty in parameter estimations. In addition, the inter-individual variability for primary PK parameters CL and V_1_ is estimated as 32.9 and 26.7%, respectively. With this level of variability, 14 subjects in the current study should be associated with a statistical power of more than 80% for primary PK parameter estimation, according to the sample size calculation recommended by the Food and Drug Administration (Wang et al., 2012). Model evaluations (diagnostic plots, bootstrap and visual predictive check) also supported the good stability and predictive performance of the final model. Monte Carlo simulation will be performed to optimize rituximab dosing in pediatric patients with FRNS/SDNS based on the developed PK model. A further clinical study with a larger sample size will be conducted to evaluate rituximab PK and the proposed new dosing scheme.

## Conclusion

In summary, rituximab PK was well described by a two-compartment model, with body surface area as significant covariate in FRNS/SDNS. The results suggest that higher doses or more frequent regimens in FRNS/SDNS may be required for optimizing therapeutic effects in this population.

## Data Availability

The original contributions presented in the study are included in the article/Supplementary Material, further inquiries can be directed to the corresponding authors.
